# Isoform‐Level Analysis Reveals Reproducible Early Changes in Transcript Usage During Human Vaccine Responses

**DOI:** 10.1002/eji.70277

**Published:** 2026-08-30

**Authors:** Lennart Riemann, Ahmed Hassan, Swantje Hammerschmidt, Anja Schimrock, Gunnar Schmidt, Nataliya Di Donato, Reinhold Förster

**Affiliations:** ^1^ Institute of Immunology, Hannover Medical School Hannover Germany; ^2^ Department of Pediatric Pneumology, Allergology, and Neonatology Hannover Medical School Hannover Germany; ^3^ Cluster of Excellence RESIST (EXC 2155) Hannover Medical School Hannover Germany; ^4^ Institute of Human Genetics Hannover Medical School Hannover Germany; ^5^ Genomics & Single‐Cell Core Unit Hannover Medical School Hannover Germany

**Keywords:** gene isoform, immune system, immunogenicity, RNA, transcriptional regulation, transcriptome, vaccination

## Abstract

Vaccine‐induced transcriptional responses have been extensively characterized at the gene level, but whether vaccination also alters transcript isoform usage remains largely unexplored. Here, we reanalyzed longitudinal whole‐blood RNA‐seq data from a discovery cohort of mRNA COVID‐19 vaccine recipients using the IsoformSwitchAnalyzeR framework and validated the findings in an independent cohort. Key findings were validated by full‐length RNA long‐read sequencing and extended to four additional vaccine cohorts covering distinct platforms and pathogens. mRNA vaccination induced a rapid and transient wave of differential transcript usage, peaking at 24 h post‐vaccination with 131 isoforms significantly altered across 107 genes, before largely resolving by Day 14. Isoform switching events were reproducible across independent cohorts and confirmed by full‐length RNA long‐read sequencing. Structural annotation of switching transcripts, including *RMI2*, *WARS1*, and *NT5C3A*, revealed changes affecting predicted protein domains and signal peptides. Notably, highly concordant isoform switching patterns were observed across MVA‐based SARS‐CoV‐2, influenza, and Ebola vaccine cohorts and showed dose‐dependent modulation. Overall, differential transcript isoform usage is a rapid and transient feature of the early human immune response to vaccination that was observed across multiple vaccine platforms. These findings reveal an underappreciated layer of transcriptional regulation that complements conventional gene‐level analyses and warrants integration into future vaccine immunogenicity studies.

## Graphical abstract

mRNA vaccination triggers an early, transient wave of altered transcript isoform usage peaking at 24 h and resolving by Day 14, affecting genes involved in innate immunity. Confirmed by long‐read sequencing and reproduced across influenza, Ebola, and MVA‐based vaccine cohorts, these results highlight a previously underappreciated feature of vaccine‐induced transcriptional regulation.

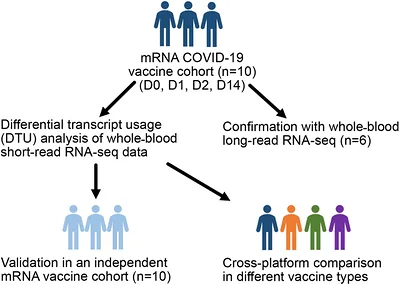

## 1 Introduction

Vaccination induces a coordinated, multilayered immune response essential for the development of protective immunity. Within hours of administration, early innate and inflammatory pathways are rapidly activated, followed by later programs of cell proliferation and differentiation [1]. Collectively, these processes shape the magnitude and quality of the subsequent adaptive immune response [1, 2, 3]. Although the underlying transcriptomic signatures of vaccination have been extensively profiled across multiple vaccine types [1, 2, 3, 4, 5], research to date has focused almost exclusively on gene‐level expression changes.

This gene‐centric framework treats each genetic locus as a uniform transcriptional unit and may, therefore, overlook the functional diversity generated by alternative splicing and alternative transcription start or termination sites. In fact, through these processes, a single gene can give rise to multiple transcript isoforms (on average 6.3 transcripts per gene locus [6]), potentially encoding proteins with distinct structural, functional and/or regulatory properties [7]. Differential transcript usage has emerged as a common and prominent feature in other biological contexts, such as cancer, where specific isoform switches have been linked to patient survival [8].

The extent to which transcript isoform changes contribute to vaccine‐induced immune responses remains largely unexplored. Characterizing isoform‐level dynamics may, therefore, add an important and previously underappreciated dimension to the molecular understanding of vaccine‐induced immunity and could reveal additional targets relevant for vaccine design. Moreover, vaccination represents a highly controlled and well‐defined immune perturbation, providing an ideal *in vivo* model to investigate the role of differential transcript usage in human immune responses more broadly.

In this study, we investigated transcript isoform dynamics in the human immune response following vaccination. By reanalyzing longitudinal RNA‐seq data from mRNA COVID‐19 vaccine recipients, we identify an early and transient occurrence of isoform changes affecting genes, such as *RMI2* and *WARS1*. These isoform‐level alterations were confirmed using full‐length RNA long‐read sequencing. Finally, we show that the same isoform signatures identified in the discovery cohort are also found across multiple other vaccine platforms, suggesting that these transcript‐level changes may extend beyond mRNA vaccination. Together, our findings highlight an additional layer of transcriptional regulation in vaccine‐induced immunity that warrants further investigation.

## 2 Results

### 2.1 mRNA Vaccination Triggers a Rapid and Transient Alteration in the Transcript Isoform Landscape

**FIGURE 1:**
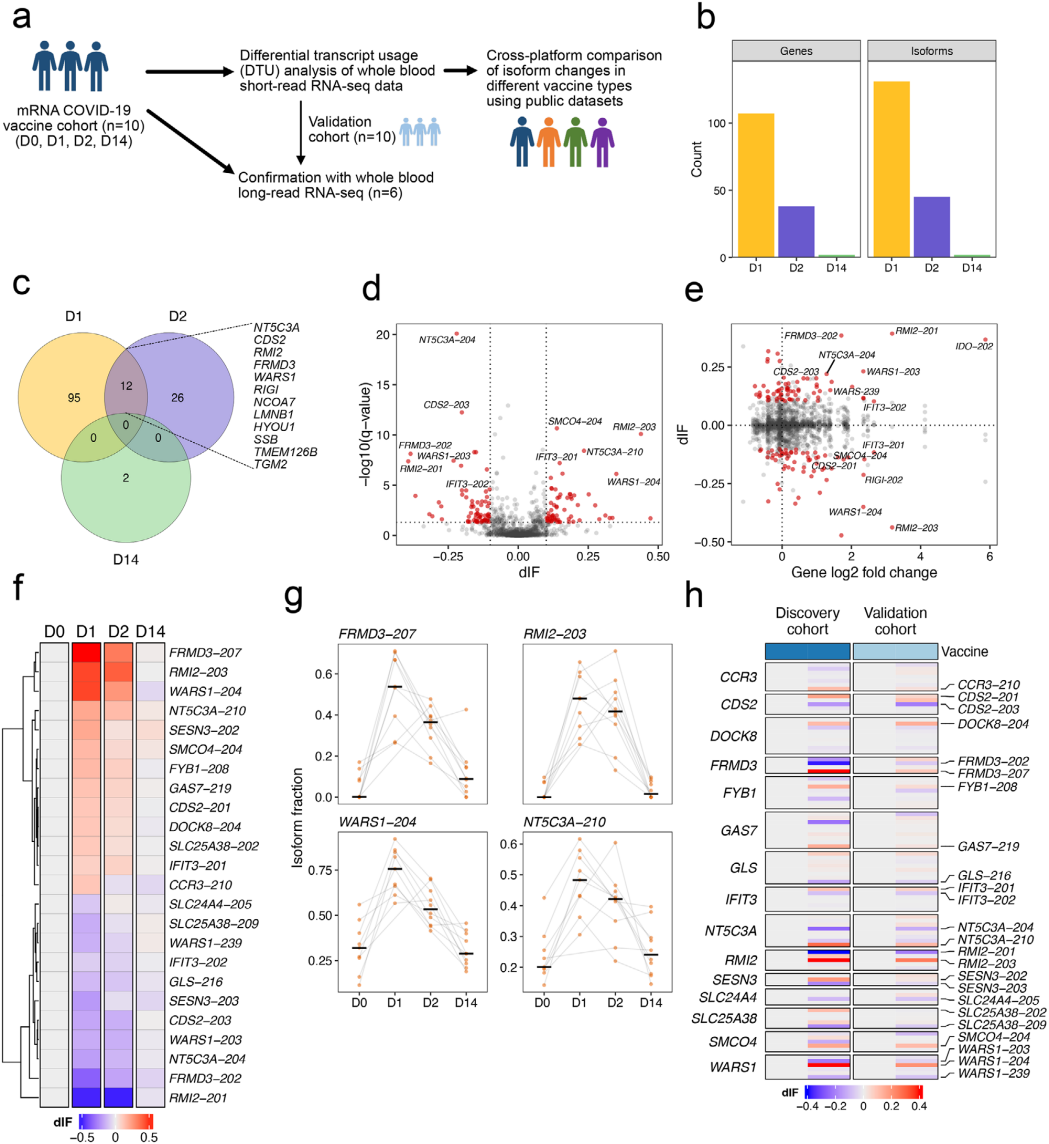
Differential transcription usage (DTU) analysis of human immune responses to mRNA COVID‐19 vaccination. (a) Study design: Schematic representation of cohorts and analysis workflow. Blood samples were taken pre‐vaccination (D0), and at Day 1 (D1), Day 2 (D2), and Day 14 (D14) post‐vaccination. (b) Summary of genes and individual isoforms exhibiting significant changes in isoform fractions at D1, D2, and D14 post‐vaccination. (c) Venn diagram illustrating the overlap of genes with significant isoform changes across the three time points. (d) Scatter plot displaying differences in isoform fraction (dIF) at D1 relative to pre‐vaccination. Isoforms with an adjusted *p* value < 0.05 and |dIF| >0.1 are shown in red. (e) Scatter plot depicting dIF values against gene‐level log2 fold changes at D1. Isoforms with an adjusted *p* value <0.05 and |dIF| >0.1 are shown in red. (f) Heatmap representing dIF values across D1, D2, and D14 relative to pre‐vaccination for the significant isoforms of the top 15 genes ranked by switching significance at D1 relative to pre‐vaccination. (g) Scatter plots showing isoform fraction dynamics for four selected isoforms: *FRMD3‐207*, *RMI2‐203*, *WARS1‐204*, and *NT5C3A‐210*. Horizontal bars represent the median. (h) Comparative heatmap showing dIF values at D1 compared to pre‐vaccination for isoforms of the top 15 genes in the discovery and validation cohorts.

Although the transcriptional response to mRNA vaccines is well‐characterized at the gene‐level—defined by a robust but transient induction of innate and interferon‐stimulated pathways—the extent to which these are accompanied by changes in transcript isoform usage remains largely unexplored. To address this, we reanalyzed longitudinal whole‐blood RNA‐seq data from a cohort of 10 individuals who received a third (booster) dose of an mRNA‐based COVID‐19 vaccine (Figure 1a), focusing on vaccination‐induced changes in transcript usage.

Utilizing the IsoformSwitchAnalyzeR framework, we quantified transcript‐level usage across four time points: baseline (D0), 24 h (D1), 48 h (D2), and 14 days (D14) post‐vaccination. This analysis revealed a pronounced wave of significant isoform variant changes immediately following vaccination. At 24 h, 131 isoforms exhibited significant changes in isoform fraction relative to baseline, mapping to 107 individual genes (Figure 1b). This response was highly dynamic and transient: by 48 h, the number of significant isoform changes decreased to 45 isoforms from 35 genes, and by Day 14, only two isoforms were significantly altered. Notably, there was only a small overlap in switching events across time points, with only 12 genes (comprising 22 significant isoforms) maintaining a significant switch from D1 to D2, all showing concordant effect directions (Figure 1c). At the 24‐h peak, substantial differences in isoform fraction (dIF) compared to baseline were observed in both increasing and decreasing directions, and in some cases occurred independently of marked changes in overall gene expression levels (Figure 1d,e).

Among the top switching isoform candidates were transcript variants originating from genes with established roles in innate immune activation and proteostasis, displaying clear yet reversible temporal dynamics (Figure 1f). For instance, specific isoforms of *RMI2* and *WARS1* underwent marked and significant shifts in isoform fraction at 24 h post‐vaccination, followed by a return to baseline by 14 days (Figure 1g). *RMI2* not only is a gene involved in DNA repair but is also associated with cell proliferation and migration [9], whereas *WARS1* encodes a tryptophanyl‐tRNA synthetase catalyzing the ligation of tryptophan to its cognate tRNA for protein synthesis [10, 11]. Importantly in the context of vaccination, *WARS1* has an additional recognized role as an innate immune activator and is an endogenous ligand for various innate receptors [10, 11].

To confirm the biological robustness of these findings, we performed an independent validation in a second cohort of 10 mRNA vaccine recipients. Isoform switching patterns for the top candidates showed a high degree of concordance in direction and magnitude of dIF changes between cohorts (Pearson *r* = 0.76, *p* < 0.001 across all isoforms; *r* = 0.87, *p* < 0.001 for significant isoforms; Figure 1h). The replication of these isoform‐specific dynamics across two independent cohorts supports isoform switching as a stable and reproducible feature of the early transcriptional response to mRNA vaccination.

Collectively, these data demonstrate that mRNA vaccination induces rapid and transient changes in transcript isoform usage, affecting multiple genes involved in early immune responses and highlighting an additional layer of transcriptional regulation beyond gene‐level expression changes.

### 2.2 Full‐Length Long‐Read Sequencing Confirms Vaccine‐Induced Isoform Alterations

Although short‐read RNA sequencing enables high‐throughput transcript quantification, it infers transcript isoforms through computational reconstruction of fragmented reads. To independently validate the vaccination‐induced isoform changes identified by short‐read sequencing, we performed full‐length RNA long‐read sequencing on a subset of participants from the discovery cohort (*n* = 6) at baseline and 24 h post‐vaccination. Given the limited number of samples, the long‐read data were used solely to confirm the direction and magnitude of isoform‐usage changes previously identified in the short‐read analysis and not to systematically identify novel isoforms.

**FIGURE 2:**
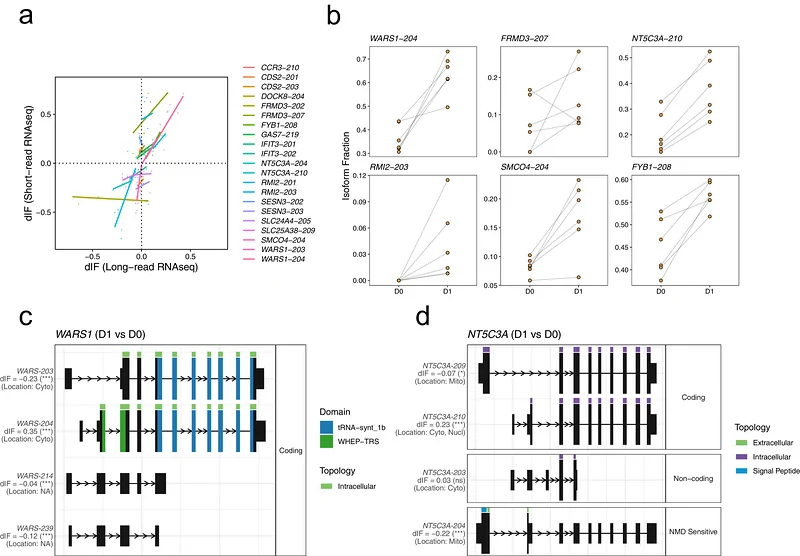
Validation of vaccine‐induced isoform changes using long‐read whole‐blood RNA sequencing (*n* = 6). (a) Concordance plot comparing dIF values from D1 relative to pre‐vaccination derived from long‐read RNA sequencing (*x*‐axis) and short‐read RNA sequencing (*y*‐axis); Pearson *r* = 0.64, *p* < 0.001 across all isoforms). (a) Concordance plot comparing dIF values from D1 relative to pre‐vaccination derived from long‐read RNA sequencing (*x*‐axis) and short‐read RNA sequencing (*y*‐axis); Pearson *r* = 0.76, *p* < 0.001 across all isoforms; *r* = 0.87, *p* < 0.001 for significant isoforms) (b) Isoform fractions D0 (pre‐vaccination) and D1 for six selected isoforms (*WARS1‐204*, *FRMD3‐207*, *NT5C3A‐210*, *RMI2‐203*, *SMCO4‐204*, *FYB1‐208*) as quantified by long‐read RNA sequencing. (c) Schematic representation of transcript structures for the annotated isoforms of the *WARS1* gene locus. (d) Schematic representation of transcript structures for the annotated isoforms of the *NT5C3A* gene locus.

Consistent with our short‐read analysis, long‐read sequencing revealed a high degree of concordance in isoform‐usage changes. Direct comparison of dIF values between the two technologies demonstrated strong directional agreement across significant isoform variants derived from the top candidate genes identified in the short‐read analysis (Pearson *r* = 0.64, *p* < 0.001 across all isoforms; Figure 2a). The vast majority of isoforms fell within the bottom‐left or top‐right quadrants of the concordance plot, indicating robustness of dIF estimates across platforms. Moreover, the magnitude and direction of isoform fraction changes of key isoforms from pre‐vaccination to 24 h post‐vaccination were reproducibly captured by long‐read sequencing (Figure 2b), providing orthogonal validation of the observed vaccine‐induced isoform alterations.

To further illustrate the potential structural consequences of vaccine‐induced switching, we visualized changes in transcript usage for two representative genes, *WARS1* and *NT5C3A* (Figure 2c,d). *NT5C3A* encodes an enzyme mediating nucleotide catabolism that is induced by interferon signaling and acts as a negative regulator of cytokine signaling [12]. For *WARS1*, the isoform that increased after vaccination (WARS1‐204) encodes an additional annotated WHEP‐TRS domain compared to the decreasing isoform (WARS1‐203), indicating that transcript usage changes affected protein‐coding regions and predicted domain composition. Similarly, for *NT5C3A*, the increasing isoform (*NT5C3A‐210*) is protein‐coding but lacks a predicted extracellular signal peptide that is present in the decreasing isoform (NT5C3A‐204), suggesting altered subcellular targeting following vaccination. Although these structural features are derived from reference transcript annotations, these examples illustrate that vaccine‐induced isoform switching can selectively modify predicted protein properties relevant to immune responses.

### 2.3 Vaccine‐Induced Isoform Switching Occurs Across Distinct Vaccine Platforms

To determine whether vaccine‐induced isoform switching is specific to mRNA vaccines or represents a more general feature of vaccine‐induced immune activation, we analyzed transcript usage for the top switching genes across publicly available short‐read RNA‐seq datasets spanning multiple vaccine platforms and pathogens.

**FIGURE 3:**
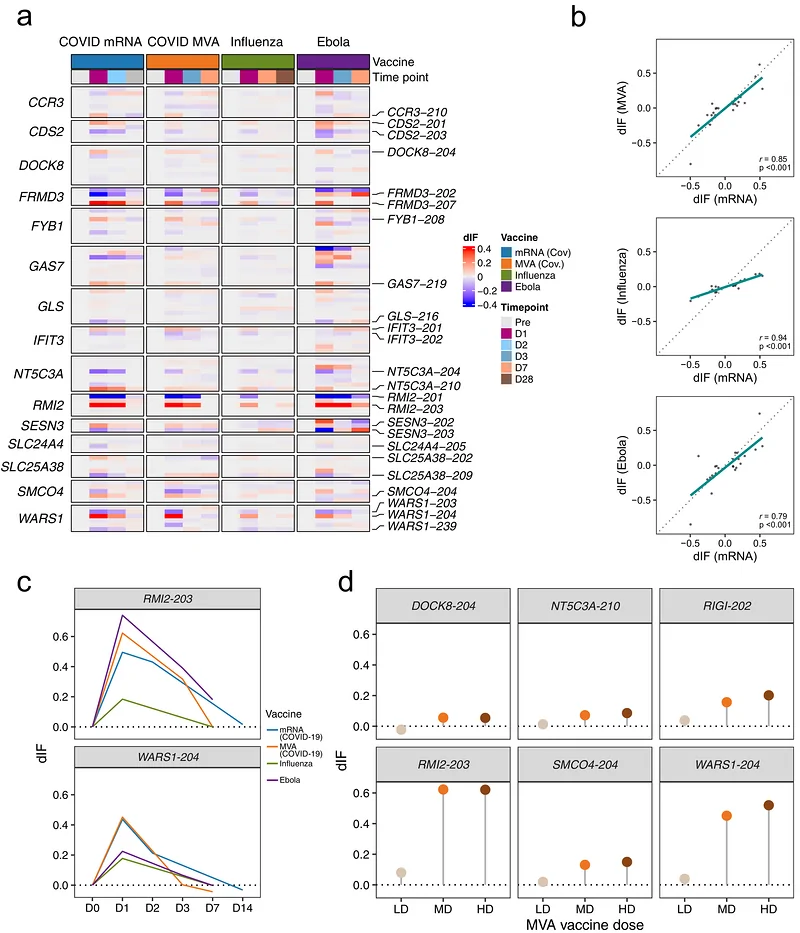
Cross‐platform comparison of differential isoform usage across distinct vaccine modalities. (a) Heatmap illustrating dIF values at indicated time points relative to pre‐vaccination across four distinct vaccine cohorts: mRNA COVID‐19 vaccine (discovery cohort, blue color bar), MVA‐based COVID‐19 vaccine candidate (medium dose, orange color bar), seasonal influenza vaccine (green color bar), and Ebola vaccine (purple color bar). Data are shown for the isoforms of the top 15 genes identified in the discovery cohort (Figure 1). (b) Correlation analyses of D1 dIF values for significant isoforms of the top 15 genes between the mRNA vaccine dataset and MVA vaccine dataset (top), mRNA vaccine and influenza vaccine datasets (middle), and mRNA vaccine and Ebola vaccine datasets (bottom). (c) Temporal dynamics of dIF values for two selected isoforms, *RMI2‐203* and *WARS1‐204*, across all four vaccine modalities. (d) Dose‐dependent comparison of D1 dIF values relative to pre‐vaccination across the low dose (LD), medium dose (MD), and high dose (HD) groups of the MVA‐based COVID‐19 vaccine candidate.

Strikingly, many of the top isoforms exhibited similar fractional shifts and transient temporal dynamics as observed in the mRNA vaccine cohort. This pattern was evident not only in a cohort receiving a different vaccine type against the same pathogen (MVA‐based SARS‐CoV‐2 vaccine candidate), but also in cohorts for a different respiratory pathogen (seasonal influenza vaccine) and a non‐respiratory pathogen (Ebola vaccine), demonstrating that these isoform changes recur across distinct vaccine platforms beyond the mRNA setting (Figure 3a). Isoforms that were not significant in the mRNA cohort remained largely unchanged across all groups, highlighting the specificity of the observed switching events.

To quantify concordance, we correlated dIF values for the top isoforms between the mRNA cohort and each of the other vaccines, revealing strong, positive correlations for mRNA versus MVA (top), mRNA versus Influenza (middle), and mRNA versus Ebola (bottom; Figure 3b). These results indicate that both the direction and magnitude of isoform changes are reproducible across vaccines, independent of pathogen or platform.

Focusing on individual transcripts previously highlighted in Figure 1g, we examined temporal dynamics for *RMI2* and *WARS1* isoforms across all four cohorts. Both isoforms showed the same transient early fractional increases as in the mRNA cohort, peaking at 24 h and returning toward baseline at later time points (Figure 3c).

Finally, to evaluate dose‐dependency, we leveraged the MVA cohort, which included low (LD), medium (MD), and high (HD) dose groups. Several key isoforms exhibited higher dIF values at MD and HD compared to LD, with the largest difference observed between LD and MD and a smaller difference between MD and HD (Figure 3d). This dose–response relationship indicates that the magnitude of isoform switching can be modulated by antigen exposure, providing additional evidence for the functional relevance of these transcript variants.

## 3 Discussion

In this study, we provide a characterization of transcript isoform dynamics in the human immune response to vaccination. Analyzing longitudinal whole‐blood RNA‐seq data across two independent cohorts of mRNA vaccine recipients, we identify a rapid and transient wave of substantial isoform switching changes at 24 h post‐vaccination, affecting genes involved in innate immune activation and cellular stress responses. These findings were independently confirmed by full‐length long‐read sequencing and were reproduced across four distinct vaccines, establishing differential transcript usage as a distinct feature of the early vaccine‐induced transcriptional response.

The transcriptomic landscape of vaccination has been extensively characterized at the gene level [1, 3, 4, 5, 13], revealing conserved signatures of innate immune activation, interferon signaling, and subsequent adaptive immune priming. However, this gene‐centric framework inherently collapses the functional diversity arising from alternative splicing and alternative transcription start or termination sites. Our data demonstrate that vaccination triggers not only changes in gene expression but also shifts in transcript isoform usage, adding a previously underappreciated regulatory layer to the vaccine response.

The cross‐platform reproducibility of isoform switching adds an important dimension to these findings. The observation of highly similar isoform dynamics following MVA‐based SARS‐CoV‐2, seasonal influenza, and Ebola vaccination suggests that these switches are not an idiosyncratic feature of mRNA vaccine formulations but rather reflect a general transcriptional response to immune activation. Together, these observations indicate that isoform switching may be consistent, stimulus‐responsive component of the early vaccine response. The orthogonal validation provided by long‐read sequencing strengthens confidence in the isoform‐level findings.

### 3.1 Data Limitations and Perspectives

Several methodological considerations warrant mention. Short‐read transcript quantification relies on computational reconstruction from fragmented reads and can misassign structurally similar transcript variants, likely underestimating true transcript usage complexity. Although concordance with long‐read sequencing supports our primary findings, the observed isoform‐switching landscape should be regarded as a short‐read‐constrained estimate rather than a comprehensive characterization of vaccination‐induced splicing events.

A further consideration concerns the repeated‐measures design: Current differential transcript usage frameworks do not readily accommodate mixed‐effect modeling, and donor identity was, therefore, not explicitly modeled as a random effect. Although this may affect statistical inference, the reproducibility across independent cohorts, sequencing technologies, and vaccine platforms supports the robustness of the observed biological patterns.

Larger studies will be needed to link specific switching events to downstream immunogenicity outcomes, whereas the predicted structural consequences identified here require experimental functional validation. Finally, because bulk whole‐blood RNA sequencing cannot resolve cell‐type specificity, and vaccination induces rapid changes in the circulating immune cell composition [2, 3], part of the observed bulk isoform alterations may reflect shifts in cell‐type proportions rather than, or in addition to, isoform changes within individual cell types. Future studies combining long‐read and single‐cell approaches will be important to distinguish cell‐intrinsic isoform changes from those driven by shifts in immune cell composition and to more comprehensively characterize vaccine‐induced transcript isoform diversity.

### 3.2 Concluding Remarks

In conclusion, this study uncovers differential transcript isoform usage as a rapid, transient, and reproducible feature of the human immune response to vaccination. By demonstrating that isoform switching occurs across vaccine platforms, which is confirmed by orthogonal sequencing technologies, and affects genes with established roles in innate immune signaling, our findings highlight an additional layer of transcriptional regulation that is not captured by conventional gene‐level analyses. Integrating isoform‐level analyses into future vaccine immunogenicity studies may deepen our understanding of the molecular mechanisms underlying vaccine‐induced immunity.

## 4 Methods

### 4.1 Cohorts

The discovery cohort has been described previously [2]. In brief, participants (*n* = 10) received a third (booster) COVID‐19 vaccination with an mRNA vaccine (BNT162b2) following heterologous priming with ChAdOx1 and BNT162b2. Although participants were originally stratified by vaccine response to the first and second vaccinations for the primary study, the present analysis focused on overall vaccine‐induced isoform dynamics. Stratified analyses were not pursued, as isoform‐level testing substantially increases model complexity and multiple‐testing burden, rendering subgroup comparisons underpowered. Only female participants were included in this cohort, as the original study design selected the largest available subgroup at the time, in order to minimize additional biological variability given the small cohort size. The validation cohort has also been described previously [2, 14] and comprised 10 vaccine recipients who received an mRNA vaccine as the booster dose, following mRNA vaccination at the second dose and either ChAdOx1 (*n* = 4) or mRNA (*n* = 6) at the first dose. This cohort included two males and eight females. Additional vaccine cohorts used for cross‐platform comparison, including an MVA‐based SARS‐CoV‐2 vaccine candidate [15], seasonal quadrivalent influenza vaccination [16], and Ebola vaccination (rVSV‐ZEBOV) [17], have been published elsewhere and were reanalyzed here using a harmonized analysis framework.

### 4.2 Samples and RNA Sequencing

Whole blood was collected from study participants (discovery and validation cohorts) into PAXgene Blood RNA tubes (QIAGEN) and processed according to the manufacturer's instructions. Tubes were gently inverted immediately after collection, incubated at room temperature for approximately 2 h, and subsequently stored at −80°C. Total RNA was extracted from thawed samples using the PAXgene Blood RNA Kit (QIAGEN) following the manufacturer's protocol and stored at −80°C until library preparation. Bulk RNA‐sequencing libraries were generated using the Illumina TruSeq mRNA library preparation protocol and sequenced according to standard Illumina workflows.

For long‐read transcriptome profiling, full‐length cDNA was prepared using Iso‐Seq Express 2.0 Kit following the manufacturer's recommendations. Twelve samples were multiplexed and used to prepare one library using Kinnex full‐length RNA kit following the manufacturer's recommendations. The library was then sequenced using the Revio system from PacBio using two wells. Sequencing results were processed and analyzed using the IsoSeq pipeline.

### 4.3 Analysis and Statistics

Isoform‐level differential transcript usage analysis was conducted using the IsoformSwitchAnalyzeR framework (v.2.6.0) in R [18]. Briefly, transcript‐level abundances were estimated from short‐read RNA‐seq data using Salmon in quasi‐mapping mode and imported into R [19, 20]. Following import, pre‐filtering was applied using a gene expression cutoff of 1, an isoform expression cutoff of 0, and an isoform fraction cutoff of 0.01, removing lowly expressed genes and those with unused/non‐contributing isoforms. GENCODE v46 was used as the reference annotation, including transcript annotations and corresponding transcript sequences (FASTA).

Differential isoform usage between baseline and post‐vaccination time points was assessed using the isoformSwitchTestDEXSeq function with a dIF cutoff of 0.05, reduceToSwitchingGenes = TRUE, and a significance threshold of 0.05 [18]. Statistical significance was determined using Benjamini–Hochberg correction for multiple testing, and isoform switches were defined on the basis of adjusted *p* values and dIF. Isoform fractions were calculated as the proportion of gene‐level expression attributable to each transcript, and dIF values were computed as the change in isoform fraction between post‐vaccination time points and baseline.

To infer potential functional consequences of isoform changes, external sequence analysis tools were used to evaluate corresponding amino acid sequences of transcript sequences. These included assessments for coding potential (CPC2 [21]), predicted protein domains (Pfam [22]), predicted signal peptides (SignalP [23]), predicted protein topology (TMHMM [24]), and predicted sub‐cellular localization of protein (DeepLoc2 [25]).

dIFs were visualized for top candidate isoforms identified in the differential usage analysis, and isoform‐level trajectories were examined across time points. Correlation analyses were performed using Pearson's correlation method. All analyses were conducted in R (v4.4.2) using publicly available software packages and established analysis frameworks (see above). Additional data of this study are available from the corresponding author upon reasonable request.

## Author Contributions

Conceptualization: Lennart Riemann and Reinhold Förster. Methodology and investigation: Lennart Riemann, Ahmed Hassan, Swantje Hammerschmidt, Gunnar Schmidt, Nataliya Di Donato, and Reinhold Förster. Data curation and formal analysis: Lennart Riemann and Ahmed Hassan. Writing – original draft: Lennart Riemann and Reinhold Förster. Writing – review and editing: Lennart Riemann, Ahmed Hassan, Swantje Hammerschmidt, Gunnar Schmidt, Nataliya Di Donato, and Reinhold Förster. Supervision: Reinhold Förster, Anja Schimrock contributed to Methodology and investigation as well as Writing – review and editing.

## Funding

The study was funded by the Deutsche Forschungsgemeinschaft (DFG, German Research Foundation) under Germany's Excellence Strategy—EXC 2155— project number 390874280.

## Ethics Statement

The primary study was approved by the ethics committees (no. 8973_BO‐K_2020 at Hannover Medical School). Additional transcriptomic datasets were obtained from the Gene Expression Omnibus (GEO) repository and analyzed as de‐identified secondary data.

## Consent

All participants provided written consent to participate.

## Conflicts of Interest

The authors declare no conflicts of interest.

## Data Availability

Raw and processed RNA‐seq datasets of the mRNA vaccine cohorts are deposited in the GEO repository under accession number GSE246525. External cohorts analyzed in this study are publicly available through GEO under accession numbers GSE247401 (second mRNA COVID‐19 vaccine cohort), GSE291862 (MVA‐SARS‐Cov2 vaccine cohort), GSE194378 (Influenza vaccine cohort), and GSE97590 (Ebola vaccine cohort). The long‐read sequencing data are deposited in GEO under accession number GSE342810. The code used for this study is publicly available on GitHub at https://github.com/lrmhh/vaccine‐isoform‐dynamics.
